# Healthcare professionals’ experiences with highly qualified nurses working in acute care teams in primary healthcare settings

**DOI:** 10.1080/02813432.2021.1913903

**Published:** 2021-04-29

**Authors:** Stine Emilie Junker Udesen, Annmarie Touborg Lassen, Nina Andersen, Christina Østervang, Dorthe Suanne Nielsen

**Affiliations:** a The Department of Elderly and Disabled, Odense Municipality, Odense, Denmark; b Department of Emergency Medicine, Odense University Hospital, Odense, Denmark; c Clinical Institute, University of Southern Denmark, Odense, Denmark; d Department of Geriatric Medicine, Migrant Health Clinic, Odense University Hospital, Odense, Denmark

**Keywords:** Health services research, qualitative research, cross-sectorial collaboration, acute nursing, acute care teams, highly-qualified nurses

## Abstract

**Objective:**

Strengthening primary healthcare with highly qualified nurses in acute care units or teams is a new Danish initiative intended to detect acute diseases and the deterioration of chronic diseases and to develop treatment for outpatients. This study explores healthcare professionals’ experiences with this initiative.

**Design:**

Qualitative semi-structured interviews conducted in 2019–2020. Analysis was conducted with a systematic text condensation.

**Setting:**

This study is based on an acute care team in one Danish municipality called Acute Team Odense (ATO). ATO delivers acute nursing in patients’ own homes (including nursing homes) in collaboration with different healthcare professionals.

**Subjects:**

Individual interviews with general practitioners (GPs) (*n* = 15), five focus-group interviews with nurses and nursing assistants from the municipality (*n* = 19) and one focus-group interview with staff from the emergency department (ED) (*n* = 10).

**Main outcomes:**

Experiences of different healthcare professionals’ experiences with ATO.

**Results:**

In general, all of the participants were very satisfied with the new acute care team and the cross-sectorial possibilities. The GPs usually referred ATO to assessments in which paraclinical equipment, competencies, accessibility, response time and communication were important. The municipal nurses and nursing assistants tended to use ATO if they needed second opinions or acute nurse assistance. The ED most often used ATO to assist with intravenous therapy after an ED visit. All participants reported that ATO increased what could be assessed and treated in patients’ homes, which is central to preventing unnecessary hospitalisations.

**Conclusions:**

ATO created new possibilities in patient’s homes which potentially might prevent unnecessary hospitalisations.

**KEY POINTS**
- Acute care units or teams are mandatory in Danish health care, but limited knowledge in the area is found.
- Healthcare professionals found that the acute care teams provided new possibilities to assess and treat patients in their own homes.
- Healthcare professionals experienced that the acute care team potentially prevented hospitalisations by fast clinical nurse assessments with paraclinical tests.

## Introduction

Worldwide, the proportion of people aged 65+ with chronic conditions is increasing; this is proving challenging for the health- and elderly-care sectors [1–3]. The needs and expectations placed on these services are growing, making it important to develop new solutions to improve the quality of health- and elderly-care services and to develop more personalised and coherent services across sectors, ensuring more value for money [1,4,5].

The prevalence of unnecessary hospitalisations and acute hospital visits is higher among older people and people with chronic conditions [6]. These groups experience a lack of consistency more often than other patients, which is why it is necessary to focus on them [2]. The Danish healthcare system is free of charge and building a strong primary healthcare sector is aimed to avoid unnecessary hospitalisations by assess and initiate treatment at home as less intrusive alternative to hospitalisations [7]. In the Danish context, general practice is responsible for medical care outside of hospitals; the regions are responsible for hospital treatment; the municipalities are responsible for home care and nursing services [8].

One alternative to admission is the mandatory new initiative from 2018 of acute care units or teams in all Danish municipalities. The initiative consists of either an acute care unit or an acute care team or both. The acute care teams are outpatient teams that provide acute nursing in patients’ own homes (including nursing homes), whereas acute care units exist in municipalities and attend to designated ‘acute beds’ [9]. These units are integrated into the country of Denmark’s strategy aimed at developing the country’s acute healthcare system. One focus area in the strategy is to increase the cross-sectorial collaboration with the goal of preventing hospitalisations if conditions can be handled in primary care settings [10].

In the acute care units and acute care teams, nurses are trained to identify acute diseases and acute deterioration of chronic diseases using systematic approaches which is the main aim of the new initiative. The acute nurses’ competencies are in place with systematic approaches known from hospital emergency departments (EDs). Other aims of these units and teams include increasing municipal nurses and nursing assistants’ competencies and further developing outpatient hospital functions [9]. Bridging primary and secondary healthcare sectors is a goal to achieve increased collaboration between municipalities, hospitals and general practice.

Thus far, limited knowledge about initiatives in the municipalities and cross-sectorial initiatives making it difficult to evaluate acute care units or acute care teams. The aim of this study is to explore healthcare professionals’ experiences with one acute care team in one Danish municipality. In this study, healthcare professionals include general practitioners (GPs), municipal nurses and nursing assistants, ED nurses and ED physicians.

## Method

### Setting

Acute Team Odense (ATO) is located in Odense, Denmark (population 205,106) [11]; it is an acute care team started in January 2018 that was designed to prevent unnecessary hospitalisations and provide treatment in adult patients’ (≥18 years) own homes (including nursing homes). ATO comprises approximately 20 nurses who are highly qualified and specialised in delivering acute nursing care in collaboration with general practice, the municipality, three departments at Odense University Hospital (OUH), the out-of-hours service and the Emergency Medical Dispatch Centre. The collaborating partners at OUH include the ED, the Geriatric Department and the Palliative Team. ATO can take different paraclinical tests and give intravenous therapy in the patient’s own homes. Patients are usually hospitalised if they need intravenous therapy. ATO is based at the ED, which is a very unique localisation that supports close cross-sectorial collaboration [12]. Usually, acute care units and acute care teams are based in the municipalities which are important to be aware of in relation to the transferability of this study.

Most of the referrals to ATO come from GPs (44%) and municipal nurses and nursing assistants (24%); only 13% of the referrals comes from OUH [13]. The last group of referrals is often more time-consuming than the others because they often require treatment at home lasting several days [13]. The remaining referrals are received from the out-of-hours service, the Emergency Medical Dispatch Centre, patients and relatives.

In this study, all of the interviews were conducted at the participants’ respective workplaces to ensure confidentiality and to save the participants travel time.

### Design

This qualitative study was inspired by a hermeneutic phenomenological approach [14]. This approach provided detailed meanings of the healthcare professionals’ experiences by understanding what they experienced and how they experienced it in relation to ATO.

### Participants

A variety of healthcare professionals was invited to participate in the study (Table 1). The rationale for interviewing different groups of healthcare professionals was to capture perspectives from different sectors that may have different perspectives and experiences towards ATO. Because most of the referrals from OUH came from the ED, nurses and physicians from the ED were selected to participate instead of interviewing staff from the other departments.

**Table 1.** Characteristics of participants.

Participants	Gender	Age^a^	Profession
**GP^b^**			
** 1**	Male	62	GP
** 2**	Male	48	GP
** 3**	Male	44	GP
** 4**	Male	41	GP
** 5**	Female	51	GP
** 6**	Female	61	GP
** 7**	Male	65	GP
** 8**	Male	55	GP
** 9**	Female	51	GP
** 10**	Male	45	GP
** 11**	Male	63	GP
** 12**	Female	45	GP
** 13**	Female	53	GP
** 14**	Male	61	GP
** 15**	Female	61	GP
**ED^c^**			
** 1**	Male	69	Physician
** 2**	Male	67	Physician
** 3**	Female	58	Physician
** 4**	Female	49	Physician
** 5**	Male	34	Physician
** 6**	Male	65	Physician
** 7**	Male	67	Physician
** 8**	Male	63	Physician
** 9**	Female	55	Nurse
** 10**	Female	49	Nurse
**Municipality^c^**			
** 1**	Female	n/a	Nursing assistants
** 2**	Female	n/a	Nursing assistants
** 3**	Female	n/a	Nursing assistants
** 4**	Female	n/a	Nursing assistants
** 5**	Female	n/a	Nursing assistants
** 6**	Female	n/a	Nurse
** 7**	Female	n/a	Nursing assistants
** 8**	Female	n/a	Nursing assistants
** 9**	Female	n/a	Nursing assistants
** 10**	Male	n/a	Nursing assistants
** 11**	Male	n/a	Nursing assistants
** 12**	Female	n/a	Nursing assistants
** 13**	Female	n/a	Nursing assistants
** 14**	Female	n/a	Nursing assistants
** 15**	Female	n/a	Nursing assistants
** 16**	Female	n/a	Nurse
** 17**	Female	n/a	Nursing assistants
** 18**	Female	n/a	Nurse
** 19**	Female	n/a	Nursing assistants

^a^Not available. It has not been possible to obtain the age of the municipal nurses and nursing assistants.

^b^Individual interviews.

^c^Focus-group interviews.

The participants were recruited by an email invitation that contained information about the study. The invitation was sent to all the GPs in Odense, the municipal leaders in Odense and to the ED’s ward management. The chairman of the GP organisation in Odense sent the invitation to the GPs. Once participants had responded, they were contacted to schedule a time and place to conduct the interviews.

### Qualitative interviews: data collection

The interviews were performed between May 2019 and January 2020 and lasted for 23–57 min. In total, 15 individual interviews were conducted with GPs (*n* = 15), five focus group interviews were conducted with municipal nurses and nursing assistants (*n* = 19) and one focus group interview was conducted with ED nurses (*n* = 2) and ED physicians (*n* = 8). All of the interviews were semi-structured and featured open-ended questions because this interview format allowed new ideas to be brought up during the interviews and supported an informal dialogue and discussion.

This choice of different interview forms was made to ensure that it was easy to participate. The participants in the focus groups were already at the same location due to their daily work while the GPs were at different and individual locations. As Breen (2006) explained, one-to-one interviews probe individual experiences, whereas focus groups provide spontaneous ideas and discussion because attitudes and opinions are socially formed [15]. The first author (SEJU) acted as a moderator in the focus group interviews, ensured that all of the participants spoke and kept the interviews on track so that all of the interview questions were answered. During the study, SEJU had been employed in the Odense Municipality as an independent researcher aimed to increase knowledge of ATO. All of the interviews were conducted in Danish. The interviews were audiotaped and transcribed. Selected quotes were translated into English.

### Interview guide

The study developed three semi-structured interview guides targeted towards the different types of participants. The rationale for developing three interview guides was that certain dimensions were not relevant for all three groups because the participants might have different interests in and approaches towards ATO. For example, some of the interview questions were only relevant for the ED staff due to treatment at home. The interview guides were developed using the literature [14] and were intended to explore the healthcare professionals’ experiences with ATO. All of the interview guides were pilot tested before the actual interviews by a group representing GPs, municipal nurses and nursing assistants and ED staff. They were all excluded from the main study after participating in the pilot tests. The pilot interviews prompted minor corrections of wordings are were not included in the final interview sample.

### Analysis

The analysis was conducted using Malterud’s systematic text condensation, which enables a descriptive, explorative and pragmatic approach [16]. The thematic analysis was not attached to a specific theoretical framework, as is the case with other qualitative methods [17]. Malterud’s analysis strategy consisted of four steps: (1) total impression – from chaos to themes, all of the transcripts were read several times to gain a data overview and to identify preliminary themes associated with the participants’ experiences with ATO; (2) identifying and sorting meaning units – from themes to codes, the transcripts were systematically reviewed to identify meaning units that were then organised into code groups; (3) condensation – from code to meaning, the meaning units were reread, and the content was reduced into condensates; (4) synthesising – from condensation to description and concepts, the content of the condensates was finally described by interpreting and understand the meaning of the condensates. Table 2 uses an example to illustrate how the method was applied.

**Table 2.** Schematic example of the steps in condensing meaning units to main themes (inspired by Malterud [16]).

Meaning unit	Condensation	Subtheme	Main theme
‘We see patients who are on the verge of requiring hospitalisation and about whom the picture is unclear; they are not able to come here’ (GP, 12).	What is the Acute Team used for?	New possibilities across sectors	New possibilities in patients’ own homes
‘The acute illness should not be spread to other morbidities – for example, if the patients need fluid or antibiotics therapy, not if the patients have a urinary tract infection and signs of delirium, or if the patients have pneumonia and lose their breath while walking’ (ED physician, 8).	The most frequent referral reasons Important factors for using ATO		
‘It gives me confidence that I have a telephone number for the Acute Team. I have the opportunity to call and talk to them directly and to get some guidance’ (Focus Group 5, municipal nurses and nursing assistants, 1). ‘I think it how it works now with a direct reference is important; they should be able to help with patient assessments’ (GP, 1). ‘The big difference is that they are based at the ED. If it was just a telephone system and we never saw the nurses, it would not have gone as well’ (ED Physician, 2).	Direct dialogues Based at the ED Close relations Competencies Available 24/7 Confidence Closer collaboration between sectors	What is important for collaboration and communication?	Collaboration and communication
‘Before the Acute Team, it was very normal to call the ED and say, “This patient sounds very bad, but I cannot see him before the afternoon”. I do not dare to wait, so he will be hospitalised’ (GP,14). ‘We identify it more quickly. People get faster treatment for pneumonia, urinary tract infections and all the small things that often result in a hospitalisation’ (Focus Group 5, municipal nurses and nursing assistants, 1). ‘As Flowmaster (a physician who manages the ED’s patient flow), you are more often contacted by the GPs when they refer a patient that ATO already has, seen and must be hospitalised after [the team’s] evaluation’ (ED Physician, 6).	More specific clinical assessments at home Seeing the patients more quickly than the GPs could GPs as gatekeepers to the ED Hospital-grade treatment at home	Contributing to preventing hospitalisations	Preventing unnecessary hospitalisations and reducing days of hospitalisation

The author group reviewed and discussed the findings of the analysis to ensure quality. The checklist consolidated criteria for reporting qualitative research (COREQ) was followed to ensure that important aspects were reported, and that transparency was achieved [18].

### Ethics

All the participants received written and verbal information about the study before the interviews were conducted. Further, all of the participants were informed that their anonymity would be ensured. Before starting the interviews, all of the participants gave informed consent to participate. The interviews and analyses were performed in accordance with the Association of Internet Researchers [19] guidelines and with Danish data protection legislation [20].

## Results

In total, 44 health professionals were interviewed. The GPs consisted of nine men and six women aged 44–65 years. The ED staff consisted of eight physicians, six men and two women aged 34–69 years and two female nurses aged 49 and 55 years. The interviewed staff from the municipality consisted of three female nurses, and 16 nursing assistants, 14 women and two men (Table 1).

The study identified the following themes as being central to all groups’ experiences with ATO by an inductive analysis strategy: (1) new possibilities in the patient’s own homes, (2) well-functioning collaboration and communication and (3) preventing potentially unnecessary hospitalisations.

### New possibilities in the patients’ own homes

Depending on the occupational backgrounds of the participants, GPs, municipal nurses and nursing assistants, ED nurses and ED physicians had different opinions about which new possibilities ATO provided. They used ATO in different ways due to their positions in the healthcare system, which explains these divergent experiences.

The GPs expressed that they most often used ATO for clinical nurse assessments of older patients with suspected acute illnesses or deterioration of chronic diseases. Several GPs also mentioned using ATO for patients who were not able to visit them due to physical impairments or other acute disease factors. A majority of the GPs explained that they were often unsure of whether patients’ medical conditions required hospitalisation: ‘We see patients who are on the verge of requiring hospitalisation and about whom the picture is unclear; these patients are not able to come into the GPs office’ (GP 12).

All of the GPs mentioned that ATO had created new opportunities for the GPs to make qualified decisions without seeing the patients in person. For example, one GP described ATO as an extension of his arms in acute situations because they could conduct clinical nurse assessments with paraclinical tests in patients’ homes. Most of the GPs expressed that it had improved the quality and breadth of what could be assessed and treated in patients’ homes: ‘I often thought that they solved it as I would do on a home visit, but with higher quality, because we do not have half of a laboratory with us when we go on home visits’ (GP 6).

The new possibilities for referring ATO were important for the GPs’ schedules in the clinic because they did not have to cancel scheduled patients to address acute situations, saving them time and reducing their acute patient home visits. Most of the GPs mentioned the following as important factors in their use: their possession of paraclinical equipment, their high level of competencies, accessibility to ATO *via* a single telephone number and ATOs fast response times.

The municipal nurses and nursing assistants shared the same opinions as the GPs regarding the new possibilities ATO had provided because of their close collaboration in the primary healthcare sector. Several of them said that the GPs were often involved when they called ATO to assess patients, as the GPs were responsible for medical care in the primary care setting. Several of the municipal nurses and nursing assistants explained that ATO had made it possible for them to get a second opinion or nurse assistance in acute situations without the need to involve GPs or the Emergency Medical Dispatch Centre. This made it easier for them to identify what was wrong with patients who had unclear symptoms: ‘If you cannot put a finger on why a patient is completely different, they can do something that I just cannot do’ (Focus Group 3, Municipal Nurse/Nursing assistant 4).

These new possibilities mentioned in the interviews could be described as upgrading the primary care setting. This description is substantiated by several municipal nurses and nursing assistants who said that ATO were the specialists in the municipality who had reduced their workloads in acute care situations.

The new possibility of referring ATO made it possible to offer hospital treatment at home to patients. Several of the ED staff explained that they most often used it for (1) intravenous therapy to patients who had an infection or were dehydrated, (2) treating patients with chronic obstructive pulmonary disease and (3) hospital follow-up (for example blood tests). The patients who were treated at home instead of at the hospital were defined as stable and able to take care of themselves by the ED physicians: ‘The acute illness of patients treated at home should not be spread to other morbidities – for example, not if the patients have a urinary tract infection and signs of delirium, or if the patients have pneumonia and lose their breath while walking’ (ED Physician 8).

Providing hospital-grade treatment at home is important to ensure a high quality of care and life for all patients. Several of the ED physicians stated that the possibilities for treatment at home became integrated into their decision-making; they saw how patients benefitted from getting treatment by ATO.

### Well-functioning collaboration and communication

The GPs, municipal nurses and nursing assistants, ED nurses and ED physicians had different perspectives regarding their collaboration and communication with ATO because they had different relationships with the team. In general, the participants had very positive attitudes of their collaboration and communication with ATO; they stated the system functioned well, even though of their different use.

It was important that the GPs could speak directly with ATO before and after they had seen a patient. One GP explained that the acute nurses were solution-orientated and spoke the same professional language as them. The direct contact enabled them to align information, expectations, paraclinical possibilities and response time. One GP expressed how helpful ATO was: ‘We are really happy about it and think that we are getting really good help. They are good at reporting back quickly. And both patients and relatives are grateful because they arrive so rapidly. It is never a problem when we call the Acute Team’ (GP 13).

The base of ATO at the ED seemed to be important for the collaboration with the ED. The physical presence made it easier and less time consuming to discharge patients to treatment at home, to make patient rounds, to hand off information and treatment equipment and to fulfil patients’ and relatives’ expectations before they were discharged: ‘The big difference is that they are based at the ED. If it was just a telephone system and we never saw the nurses, it would not have gone so well’ (ED Physician 2).

A few of the ED physicians also said that the patients and relatives were very satisfied to see an acute nurse from ATO before being discharged. Several of the acute nurses had previously worked at the ED, which improved collaboration and confidence in terms of discharging patients to them: ‘I feel extremely confident that the nurses from the Acute Team take out and assess these patients. They have the same credibility as the nurses in the ED’ (ED Physician 6).

The municipal nurses and nursing assistants could call ATO directly 24/7 if they needed a second opinion or help in an acute situation. This gave them extra confidence during their daily work, boosting their collaboration and communication with ATO. For example, a primary health professional said: ‘It gives me confidence that I have a telephone number for the Acute Team. I have the opportunity to call and talk to them directly and get some guidance’ (Focus Group 5, Municipal Nurse/Nursing assistant 1).

Another important aspect of this collaboration was the GPs’ involvement because they played a central role in prescribing paraclinical tests. A majority of the municipal nurses and nursing assistants found that ATO closely collaborated with GPs and ED physicians, further creating new possibilities for care. For example, they said that GPs now initiated treatment more quickly. The collaboration also contributed to new solutions in acute situations, allowing to help patients who urgently needed special equipment: ‘The hospital is involved, which contributes to new solutions. Before, I just thought it was a shame when I couldn’t get certain equipment to patients’ (Focus Group 3, Municipal Nurse/Nursing assistant 1).

### Preventing potentially unnecessary hospitalisations

The new solution of referring ATO to patients became critical to different healthcare professionals’ ability to prevent unnecessary hospitalisations. Due to their different positions, GPs, municipal nurses and nursing assistants, ED nurses and ED physicians all had different options to prevent potentially unnecessary hospitalisations.

As mentioned, the GPs played a central role in preventing unnecessary hospitalisations. Before ATO was established, many of the interviewed GPs had been forced to hospitalise patients because they did not have the time to go on acute home visits and/or because they did not have the same paraclinical equipment as them: ‘Before the Acute Team, it was very normal to call the ED and say, “This patient sounds very bad, but I cannot see him before the afternoon”. I do not dare to wait, so he will be hospitalised’ (GP 14).

The ability to take blood tests were especially important for preventing potentially unnecessary hospitalisations, as they could be used to confirm or exclude infection or dehydration: ‘We cannot get an exact diagnosis from home visits because we cannot see how dehydrated or infected someone is; thus, we must hospitalise them. But when we are told that their fluid balance is fine or their level of infection and that they can breathe, then we know it is okay, and we can give them antibiotics while they remain at home’ (GP 6).

The municipal nurses and nursing assistants also played an important role in preventing unnecessary hospitalisation because they collaborated with the GPs and called ATO if they needed help in situations that could be handled in the primary setting. Several of them stated that ATO often prevented hospitalisations due to suspected infection or dehydration, trouble placing urinary catheters or in other situations that required help: ‘We identify it more quickly. People get faster treatment for pneumonia, urinary tract infections and all the small things that often result in a hospitalisation’ (Focus Group 5, Municipal Nurse/Nursing assistant 1).

The ED nurses’ and ED physicians’ perspectives on preventing unnecessary hospitalisations focussed on how they experienced changes in the GPs’ behaviour after ATO was established. They found that ATO acted like gatekeepers to the ED. One ED physician expressed his experiences with the GPs: ‘As Flowmaster (a physician who manages the ED’s patient flow), you are more often contacted by the GPs when they refer a patient that ATO already has seen and must be hospitalised after ATOs evaluation’ (ED Physician 6).

As a supplement to preventing potentially unnecessary hospitalisations, ATO reduced the length of hospitalisations. Most of the patients, the ED discharged to ATO would previously have been hospitalised with intravenous therapy for several days. One ED physician explained that ATO made it possible to offer patients the same or better treatment at home: ‘Some patients would probably have been discharged a little earlier with oral antibiotics. So, there are some patients who get a slightly better treatment than they would have received otherwise. Others get the same treatment, but at home’ (ED Physician 2).

## Discussion

### Statement of principal findings

This study explored different healthcare professionals’ experiences with the new acute care team ATO. The GPs usually referred ATO to do assessments in which paraclinical equipment, competencies, accessibility, response time and direct communication were important. The municipal nurses and nursing assistants tended to use it if they need second opinions or acute nurse assistance. The ED most often used it to assist with intravenous therapy at home after an ED visit. All participants reported that ATO increased what could be assessed and treated in patients’ homes and that the new possibilities potentially prevented unnecessary hospitalisations.

### Strengths and weaknesses of the study

The study provides insight into different healthcare professional perspectives which is considered a strength of the study. The patients’ and relatives’ perspectives on ATO are also important, but they were outside the scope of this study. The healthcare professionals were invited to participate by different methods which might have implications like risk of selection bias among the GPs. The GPs who participated might have special interest towards ATO which could have importance for the findings. The study is not designed to cover either benefits or drawbacks of ATO but to explore the participant’s experiences by an inductive analysis strategy. Our findings showed that ATO was evaluated very positive from all user perspectives. This might be a result of an interview guide not focusing on the drawbacks of ATO. Changes in the interview guide could have added this perspective and elaborated the results.

The individual interviews with the GPs were used instead of focus group interviews because of logistical challenges. The individual interviews provided insight into more personal experiences and perceptions of ATO than the focus group interviews provided. In respect of a busy organisation, we chose focus group interviews with the municipal nurses and nursing assistants and ED staff as they gather lots of information in a short time. The study acknowledges that traditionally, 10 participants in a focus group interview is too many [15]; that said, the moderator ensured that all of the participants spoke during the interviews, reporting that all of the focus groups were homogeneous and dynamic. Theoretically, different professions and statuses could have a negative influence on dialogues within focus groups [21], but this did not seem to be applicable in the present work; the hierarchical relationship [22] between the nurses and physicians did not affect open and spontaneous dialogues. The missing information of the municipal nurses and nursing assistants’ age is considered as a limitation together with the missing information of the healthcare professionals’ years of professional experience.

Different interview formats could be considered as a weakness because different types of interviews give different information. Focus group participants do not have the same opportunities to share individual experiences, whereas individual interviews do not challenge participants’ perspectives to the same degree as focus groups do [15].

The author team has attempted to be transparent about how the study was conducted, which is important for its internal validity. The mix of authors and discussion with other collaborating partners challenged the authors’ pre-understandings, which is considered to be a strength because it made the reflection and interpretation processes more thorough.

### Findings in relation to other studies

Establishing municipal acute care units or acute care teams are aimed at strengthening the primary healthcare sector and developing outpatients treatment [9]; unfortunately, evidence about the importance of this in Danish acute healthcare system does not exist.

All of the healthcare professionals’ experiences are in line with the Danish National Board of Health’s written guidelines [9] for what this initiative should provide in collaboration with general practice, municipalities and hospitals. What the different acute care units or acute care teams provide exactly depends on the individual municipality [12]. The localisation of ATO at the ED is very unique and was described as an important factor for the collaboration. This helped to create a well-functioning system because they had become familiar with each other’s organisational procedures, perceptions of how to treat the patients, work cultures and roles. These aspects are important for interprofessional collaboration [22–24] which might help to provide acute and personalised nursing in patients’ homes. As Morley and Cashel found, perceived benefits are important for the collaboration [25]. The participants experienced different benefits from using ATO. The ED staff reported that it reduced days of hospitalisation and that patient’s benefit by getting treatment at home. The GPs found that it reduced the burden of acute home visits and the municipal nurses and nursing assistants reported that it reduced workload. A Danish study found that municipal nurses were challenged by acute situations [26] which support that acute care teams added requested competencies.

This study revealed that ATO’s collaborating partners agreed that the acute care team contributed with new possibilities. The GPs reported that before ATO, they had often been forced to hospitalise patients because they could not achieve the same response time or offer the same tests as them. This reveals GPs’ role in giving patients alternatives to hospitalisation [7]. A study from UK reported that GPs were sometimes forced to hospitalise patients because they wanted to respond to the patients’ conditions quickly and that unnecessary hospitalisations could be impeded by missing communications across sectors, lack of resources or missing competencies in the primary setting [27]. The Danish study also found that nurses faced obstacles caring for patients due to missing competencies [26]. These studies support the present findings about the importance of cross-sectorial collaboration and the substantiates for solution like acute care teams. Evidence about how different initiatives improve cross-sectional collaboration is limited. One Danish study found that one municipal acute care team increased the cross-sectorial collaboration [28] which is in line with the present study. In Norway, they have municipal acute units that are different from the Danish model. A Norwegian study found that the cross-collaboration was challenging because healthcare professionals had trouble with sharing information across sectors and different perceptions of which patients were candidates to stay at these units [29]. This study found the opposite to be true because the collaboration was going well, the main group of patients seemed to be clear and information exchange protocols were clear. The divergent findings between studies might be caused by differences in setting; the Norwegian acute care units are established as acute beds at fixed locations and not as outgoing teams. Another Norwegian study found that healthcare professionals remained unsure about the appropriateness of the municipal acute care units [30]. In contrast, the participants in this study were satisfied with the new initiative. The localisation at the ED might have improved satisfaction and facilitated close and dynamic collaboration. It is important to acknowledge that most of the Danish municipal acute care teams are located without direct access to ED’s why they do not have the same possibilities. It might have importance for the cross-sectorial collaboration and the transferability of this study.

### Implications for health policy and future research

Knowledge about what happens in primary care settings and across sectors is limited which makes this study valuable. The healthcare professionals in this study reported that their collaboration with ATO created new possibilities to assess and treat patients at home, which is important for developing outpatient hospital functions and improving acute healthcare services across sectors. The findings of this study can also be used for quality development in the field and to explore inhibiting and promoting factors regarding the implementation of acute care teams. In this context, it is important to be aware of organisational- and sociodemographic differences among municipalities, as this might be important for the establishments. The present findings can be used to inspire other municipalities and collaborators to development of acute services. For future studies, it could be relevant to interview out-of-hours GPs and to prove whether the acute care teams prevent unnecessary hospitalisations.

## References

[1] National Institute on Aging, National Institutes of Health, U.S. Department of Health and Human Services. Global health and aging. Report No: 11–7737. Geneva (Switzerland): World Health Organization; 2011.

[2] Frølich A, Olesen F, Kristensen I. Hvidbog om multisygdom: dokumentation af multisygdom i det danske samfund: fra silotaenkning til sammenhaeng (White Paper on multi-disease: documentation of multi-disease in Danish society: from silo-thinking to context). Denmark: Fjerritslev Forlag; 2017.

[3] Barnett K, Mercer SW, Norbury M, et al. Epidemiology of multimorbidity and implications for health care, research, and medical education: a cross-sectional study. Lancet. 2012;380(9836):37–43. 22579043 10.1016/S0140-6736(12)60240-2

[4] World Health Organization. Strategy and action plan for healthy ageing in Europe, 2012–2020. Geneva (Switzerland): World Health Organization; 2012.

[5] Busse R, Klazinga N, Panteli D, et al. Improving healthcare quality in Europe: characteristics, effectiveness and implementation of different strategies. Paris (France): OECD Publishing; 2019. 31721544

[6] The Danish Ministry of Health. Sundheds- og aeldreøkonomisk analyse: kontaktmønstre på tvaers af sektorer blandt befolkningen, kronikere og aeldre medicinske patienter (Health and geriatric economic analysis: contact patterns across sectors among the population, chronic patients and elderly medical patients). Sundheds- og AEldreministeriet; Copenhagen Denmark: The Danish Ministry of Health; 2018.

[7] Møller Pedersen K. Kommunal medfinansiering og kampen for at forebygge indlaeggelser: viden om effekt. Økonometrisk analyse (Municipal co-financing and the fight to prevent hospitalizations: knowledge about effect. Econometric analysis). Report No.: 1. Odense. Denmark: Departement of Business and Economics, University of Southern Denmark; 2019.

[8] Healthcare in Denmark: an overview. New Delhi (India): Ministry of Health; 2016.

[9] National Board of Health (Denmark). Kvalitetsstandarder for kommunale akutfunktioner i hjemmesygeplejen – krav og anbefalinger til varetagelse af saerlige sygeplejeindsatser (Quality standards for municipal acute care in home nursing – requirements and recommendations for handling acute care units) [Internet]. København: Sundhedsstyrelsen; 2018. Available from: https://www.sst.dk/da/udgivelser/2017/∼/media/F69BEB14789842818FA1096DE20C19D9.ashx.

[10] Regions of Denmark. Når du har brug for os - 24 nye indsatser når du bliver akut syg eller kommer til skade (When you need us - 24 new efforts when you become acutely ill or injured). Denmark: Regions of Denmark; 2018.

[11] Statistics Denmark [Internet]. Statbank population. Elections. 2020. [cited 2020 Jul 2]. Available from: https://www.statbank.dk/statbank5a/default.asp?w=1366

[12] Carve Consulting. DATAUNDERSTØTTELSE AF KOMMUNALE AKUTFUNKTIONER [Internet]. 2019. Available from: https://www.kl.dk/media/20848/dataunderstoettelse-af-kommunale-akutfunktioner.pdf

[13] Udesen SEJ. Akutteam Odense Årsrapport 2019: PIXIUDGAVE (annual report 2019: Pixi Edition) [Internet]. 2019. Available from: https://www.odense.dk/-/media/images/borger/sundhed-og-sygdom/sygepleje/akutteam/aarsrapport-for-akutteam_2019.pdf?la=da

[14] Skovdal M, Cornish F. Qualitative research for development: a guide for practitioners. Rugby (UK): Practical Action Publishing Ltd; 2015. [cited 2019 Mar 21]. Available from: https://www.developmentbookshelf.com/doi/book/10.3362/9781780448534

[15] Breen RL. A practical guide to focus-group research. J Geogr High Educ. 2006;30(3):463–475.

[16] Malterud K. Systematic text condensation: a strategy for qualitative analysis. Scand J Public Health. 2012;40(8):795–805. 23221918 10.1177/1403494812465030

[17] Braun V, Clarke V. Using thematic analysis in psychology. Qual Res Psychol. 2006;3(2):77–101.

[18] Tong A, Sainsbury P, Craig J. Consolidated criteria for reporting qualitative research (COREQ): a 32-item checklist for interviews and focus groups. Int J Qual Health Care. 2007;19(6):349–357. 17872937 10.1093/intqhc/mzm042

[19] Association of Internet Researchers. Internet research: ethical guidelines 3.0 association of internet researchers [Internet]. 2019. Available from: https://aoir.org/reports/ethics3.pdf.

[20] EU. Europa-Parlamentets og Rådets, fordning nr. 2016/679 om beskyttelse af fysiske personer i forbindelse med behandling af personoplysninger og om fri udveksling af sådanne oplysinger (databeskyttelsesfordningen) (Data protection regulations of European Parliament). Brussels, Belgium: EU; 2016.

[21] Tausch AP, Menold N. Methodological aspects of focus groups in health research: results of qualitative interviews with focus group moderators. Glob Qual Nurs Res. 2016;3:2333393616630466. 28462326 10.1177/2333393616630466 PMC5342644

[22] Fewster-Thuente L, Velsor-Friedrich B. Interdisciplinary collaboration for healthcare professionals. Nurs Adm Q. 2008;32(1):40–48. 18160862 10.1097/01.NAQ.0000305946.31193.61

[23] Martin J, Ummenhofer W, Manser T, et al. Interprofessional collaboration among nurses and physicians: making a difference in patient outcome. Swiss Med Wkly [Internet]. 2010. [cited 2020 Jul 29]; Available from: http://doi.emh.ch/smw.2010.13062 10.4414/smw.2010.13062 20458647

[24] Schot E, Tummers L, Noordegraaf M. Working on working together. A systematic review on how healthcare professionals contribute to interprofessional collaboration. J Interprof Care. 2020;34(3):332–342. 31329469 10.1080/13561820.2019.1636007

[25] Morley L, Cashell A. Collaboration in health care. J Med Imaging Radiat Sci. 2017;48(2):207–216. 31047370 10.1016/j.jmir.2017.02.071

[26] The Danish Center for Social Science Research. Kompleksitet i den kommunale hjemmepleje: analyse af sygeplejerskernes perspektiv på kompleksitet i sygeplejen: rapport (Complexity in municipal home care: analysis of the nurses’ perspective on complexity in nursing). Copenhagen, Denmark: The Danish Center for Social Science Research; 2018.

[27] Hammond CL, Pinnington LL, Phillips MF. A qualitative examination of inappropriate hospital admissions and lengths of stay. BMC Health Serv Res. 2009;9:44. 19265547 10.1186/1472-6963-9-44 PMC2655293

[28] The Danish Center for Social Science Research. Tvaergående akutfunktion mellem Esbjerg Kommune og Sydvestjysk Sygehus – Organisering, oplevede resultater, effekter og økonomi (Acute care team across Esbjerg Municipality and Hospital South West Jutland - Organization, experienced results, effects and finances) [Internet]. 2019. Available from: https://www.vive.dk/media/pure/11983/2444001

[29] Johannessen AK, Steihaug S. Municipal acute units as part of the clinical pathway for older patients. Int J Integr Care. 2019;19(4):2. 10.5334/ijic.4643 PMC6823773 31736678

[30] Johannessen A-K, Steihaug S. The function of the Norwegian municipal acute units fails to fulfill the intention of health authorities. Scand J Prim Health Care. 2020;38(1):75–82. 31980001 10.1080/02813432.2020.1717085 PMC7054966

